# Radial Organization in the Mammalian Nucleus

**DOI:** 10.3389/fgene.2020.00033

**Published:** 2020-02-12

**Authors:** Nicola Crosetto, Magda Bienko

**Affiliations:** Science for Life Laboratory, Department of Medical Biochemistry and Biophysics, Karolinska Institutet, Stockholm, Sweden

**Keywords:** 3D chromatin architecture, gene expression regulation, nucleus, genome organization, chromosoma

## Abstract

In eukaryotic cells, most of the genetic material is contained within a highly specialized organelle—the nucleus. A large body of evidence indicates that, within the nucleus, chromatinized DNA is spatially organized at multiple length scales. The higher-order organization of chromatin is crucial for proper execution of multiple genome functions, including DNA replication and transcription. Here, we review our current knowledge on the spatial organization of chromatin in the nucleus of mammalian cells, focusing in particular on how chromatin is radially arranged with respect to the nuclear lamina. We then discuss the possible mechanisms by which the radial organization of chromatin in the cell nucleus is established. Lastly, we propose a unifying model of nuclear spatial organization, and suggest novel approaches to test it.

## The Building Blocks of Nuclear Architecture

The nucleus is a sub-cellular organelle that has evolved to enable the storage, preservation, reading, and duplication of the information encoded in the DNA sequence. Evidence collected over the past 50 years strongly suggests that this functional specialization is made possible by a multi-level spatial organization that manifests itself at various length scales. The nuclear space is filled by both chromatin and sub-nuclear structures—including nucleoli (Németh and Längst, 2011), nuclear speckles (Spector and Lamond, 2011), and various types of nuclear bodies (Mao et al., 2011)—that contribute to orchestrate genomic functions in various ways. The linear genomic sequence is organized into structural domains that form the building blocks of the higher-order three-dimensional (3D) architecture of the genome. Chromosomes typically condense into distinct masses known as chromosome territories (CTs), whose existence was proposed already more than a century ago by the Austrian anatomist Carl Rabl and the German biologist Theodor Boveri (Strickfaden et al., 2010), and later confirmed in multiple cell types and species (Cremer and Cremer, 2001; Cremer et al., 2006). At the sub-chromosomal level, structural domains comprise megabase (Mb)-sized cytobands visible in metaphase chromosomes, and A/B compartments identified by Hi-C (Lieberman-Aiden et al., 2009), as well as smaller domains spanning from several kilobases (kb) up to a few Mb, including topologically associating domains (TADs) (Dixon et al., 2012) and long-range chromatin loops (Rao et al., 2014). A and B compartments are defined as genomic regions that tend to engage in homotypic (A-A and B-B) rather than heterotypic (A-B) contacts, and largely overlap with euchromatic and heterochromatic cytobands, respectively (Lieberman-Aiden et al., 2009). Each A/B compartment consists, in turn, of a variable number of TADs, defined computationally as clusters of increased contacts between adjacent parts of the same chromosome in the Hi-C data matrix (Dixon et al., 2012). TADs represent hubs of *cis*-interactions often contained within chromatin loops anchored by CCCTC-binding factors (CTCF) (Rao et al., 2014), which facilitate specific enhancer-promoter contacts, while preventing unspecific and potentially detrimental interactions (Rowley and Corces, 2018). Accordingly, disruption of specific TAD borders and chromatin loops results in aberrant gene expression and can cause a variety of disease conditions, including developmental defects and cancer (Corces and Corces, 2016; Spielmann et al., 2018). TAD borders are highly conserved across metazoans and tend to coincide with dense clusters of conserved noncoding elements (Harmston et al., 2017), whereas A/B compartments appear to be less conserved across cell types and species (Schmitt et al., 2016a; Szabo et al., 2019; Yang et al., 2019). B compartments overlap, to a large extent, with lamina-associated domains (LADs), defined as genomic regions that frequently contact the nuclear lamina (Guelen et al., 2008). Depending on the cell type, LADs may comprise up to one third of the whole genomic sequence, and many LADs overlap with nucleoli-associated domains (NADs) (Németh et al., 2010) as well as with pericentromeric heterochromatin-associated domains (PADs) (Wijchers et al., 2015), suggesting that a substantial part of the genome is either localized at the nuclear periphery, close to the lamina, or in the inner part of the nucleus, around nucleoli. Unlike CTs, A/B compartments, TADs, and LADs were originally identified using bulk assays that average the signal over millions or even billions of cells. However, the recent development of single-cell Hi-C (Nagano et al., 2013; Flyamer et al., 2017; Ramani et al., 2017; Stevens et al., 2017; Ramani et al., 2020), Dip-C (Tan et al., 2018), and single-cell DamID (Kind et al., 2015), together with super-resolution microscopy assays (Boettiger et al., 2016; Wang et al., 2016; Bintu et al., 2018; Nir et al., 2018), have made it possible to confirm the existence of these sub-chromosomal domains in single cells. Another important consideration is that, although A/B compartments, TADs, and LADs have been observed in different cell types and species, all of the studies conducted so far have used either *in vitro* cultured cells or cells freshly dissociated from their tissue of origin. Therefore, we still lack a comprehensive portrait of the sub-chromosomal organization in cells directly embedded in their tissue context. Achieving this will require the development of novel assays combining high-throughput sequencing with preservation of spatial information.

Independently of the length scale at which chromatin domains are observed, studying how they are spatially arranged in the cell nucleus requires the definition of a reference system of coordinates. Since individual nuclei have different shapes and lack defined symmetry axes, one approach is to measure the distance of different chromatin domains from each other or from well-defined sub-nuclear structures serving as reference, such as the nuclear lamina. This has been classically achieved through the use of microscopy techniques, such as DNA fluorescence *in situ* hybridization (FISH), which allows measuring the distance of chromosomes or individual genomic loci from each other or from defined nuclear structures, in single cells. More recently, a new method named TSA-seq was developed to infer the relative distance from nuclear speckles of thousands of genomic loci simultaneously, based on next-generation sequencing (Chen et al., 2018). However, unlike DNA FISH, TSA-seq is a bulk assay that averages the signal over millions of cells, and thus, at least in its current design, cannot provide spatial information at the single-cell level.

In this review, we primarily focus on studies that have assessed the radial position of individual chromosomes or smaller chromatin domains relative to the nuclear periphery and center—which we here refer to as “chromatin radiality.” For a detailed description of the folding principles of chromatin in the nucleus, of the available methods for mapping 3D genome architecture, and of the role of 3D genome organization in physiological and pathological processes, we instead refer the reader to many excellent recent reviews that have extensively covered these topics (Bonev and Cavalli, 2016; Corces and Corces, 2016; Dekker and Mirny, 2016; Schmitt et al., 2016b; Rowley and Corces, 2018; Zheng and Xie, 2019).

### Radial Arrangement of Chromosomes

One of the best studied aspects of chromatin radiality is how individual CTs or selected gene loci are arranged with respect to the nuclear lamina. Early studies that examined the location of chromosomes in metaphase spreads prepared from cultured human fibroblasts, found that larger chromosomes were generally more peripherally located compared to smaller ones (Ockey, 1969; Hoo and Cramer, 1971). These observations were subsequently recapitulated in interphase nuclei of different human cell types, in which the nuclear lamina is preserved, revealing that the radial position of CTs with respect to the lamina is associated with the size of the chromosomes in base-pairs, but also with the density of genes along each chromosome (Manuelidis, 1985; Lichter et al., 1988; Nagele et al., 1999; Bridger et al., 2000; Sun et al., 2000; Boyle et al., 2001; Mahy et al., 2002; Weierich et al., 2003; Bolzer et al., 2005; Wiblin et al., 2005; Grasser et al., 2008; Jowhar et al., 2018a). Accordingly, despite having a very similar size, chromosomes (chr) 18 and 19 are mostly localized at the periphery and center of human interphase nuclei, respectively (Croft et al., 1999). Similar findings were also reported for primates (Tanabe et al., 2002; Tanabe et al., 2005; Mora et al., 2006), mouse (Parada et al., 2004; Mayer et al., 2005), and other vertebrate species (Federico et al., 2006; Skinner et al., 2009). In contrast, the radial position of CTs appears less defined in plant cells (Pecinka et al., 2004), although a tendency for centromeres to be closer to the nuclear lamina and telomeres to be more central was observed (Schubert et al., 2012; Schubert et al., 2014), which is reminiscent of the pattern of centromeres and telomeres in human and mouse cells (Weierich et al., 2003). In dividing cells, the 3D genome architecture is massively remodeled at every mitosis, and then re-established at the onset of the subsequent G1-phase, remaining relatively stable until the next mitosis (Manders et al., 1999; Edelmann et al., 2001; Lucas and Cervantes, 2002; Walter et al., 2003; Nagano et al., 2017; Gibcus et al., 2018). However, changes in the radial position of CTs and individual gene loci can occur in a variety of physiological conditions, including cell differentiation (Kuroda et al., 2004; Stadler et al., 2004; Marella et al., 2009a; Sehgal et al., 2016; Orsztynowicz et al., 2017), gametogenesis (Scherthan et al., 1998; Mudrak et al., 2009), signaling in response to extra-cellular stimuli (Branco et al., 2008; Mehta et al., 2010; Mourad et al., 2014; Ioannou et al., 2015), as well as following DNA damage (Spitkovsky et al., 2002; Mehta et al., 2013; Schwarz-Finsterle et al., 2013; Kulashreshtha et al., 2016). Importantly, the radial placement of CTs in the nucleus is often altered in cancer cells (Cremer et al., 2003; Murata et al., 2007; Marella et al., 2009b; Timme et al., 2011) and in the presence of chromosomal translocations and aneuploidies associated with cancer (Taslerová et al., 2003; Taslerová et al., 2006; Harewood et al., 2010; Allinne et al., 2014) or congenital disorders (Jowhar et al., 2018b; Kemeny et al., 2018). Altogether, these findings suggest that the non-random radial arrangement of chromosomes and sub-chromosomal regions with respect to the nuclear lamina is a universal feature of nuclear architecture, which is conserved across species and whose alteration is associated with a variety of disease conditions. It should be noted, however, that the observation that CTs and gene loci have a preferred radial location must be interpreted probabilistically, meaning that a given chromosome or locus will never be found at the same radial distance from the nuclear lamina and have the same shape or orientation in all the cells examined. Indeed, a recent study based on high-throughput DNA FISH, which measured the position of hundreds of genomic loci in thousands of human fibroblast cells, revealed that the radial distance of the same locus from the nuclear lamina is highly variable across cells (Finn et al., 2019). Thus, although individual CTs and specific gene loci have a clear propensity for being localized closer or farther from the nuclear lamina, there is a high cell-to-cell variability in the radial placement of chromatin in the nucleus. Another important consideration is that, although the non-random radial organization of CTs has been well documented in many cell types and different species, until recently only a few studies have examined CTs in cells in their natural tissue context (Solovei, 2010; Kernohan and Bérubé, 2014; Fields et al., 2019). In the future, it will be important to extend these studies to explore how the spatial arrangement of chromosomes in the nucleus is affected by cell identity, nuclear shape, surrounding cell types, and the geometry of the tissue, in different tissues and organs.

In addition to being radially arranged, several lines of evidence indicate that CTs have a non-random internal structure that is also radially organized. Early studies by DNA FISH in cultured human, mouse, and Chinese hamster cells, showed that CTs have a polarized structure, with gene-rich regions more centrally located compared to gene-poor parts of the same chromosome (Sadoni et al., 1999; Küpper et al., 2007). In human lymphocytes, telomeres were found to be, on average, closer to the nuclear center compared to centromeres, and q-telomeres were more central compared to the p-telomeres of the same chromosome (Amrichová et al., 2003). More recently, the existence of a polarized structure of individual CTs was confirmed in two studies that used DNA FISH to visualize either multiple TADs or LADs together with inter-LAD regions belonging to the same chromosome (Wang et al., 2016; Luperchio et al., 2018). These single-cell studies revealed that TADs belonging to A and B compartments (A- and B-TADs, respectively) were spatially polarized in most of the cells analyzed (Wang et al., 2016), and that, within the same chromosome, LADs and inter-LAD (iLAD) regions were clearly radially separated (Luperchio et al., 2018). One limitation of these studies is that only a few chromosomes were investigated in cultured cells of a single cell type (mouse embryonic fibroblasts in (Luperchio et al., 2018) and human fibroblasts in (Wang et al., 2016)). In the future, application of high-throughput FISH techniques, together with novel ways for visualizing the internal structure of CTs, such as ‘chromosome spotting’ (Gelali et al., 2019), will enable us to draw a refined portrait of the internal radial organization of all chromosomes, in many different cell types.

### Nuclear Periphery *vs.* Center

Aside from the notion that CTs are radially positioned in the nucleus and have a polarized internal structure, the only other aspect of chromatin radiality that is relatively well understood is the fact that the chromatin adjacent to the nuclear lamina is structurally and functionally different from the chromatin found in more centrally located parts of the nucleus. Early investigations of the nuclear structure of chicken erythrocytes by electron microscopy revealed the presence of large blocks of electron-dense material aligned all along the nuclear lamina, whereas more interior regions appeared significantly less dense (Davies, 1968; Everid et al., 1970). The electron-dense chromatin in the nuclear periphery corresponds to the LADs identified in human fibroblasts using lamin DamID (Guelen et al., 2008), and is enriched in LINE repeats and several histone marks of transcriptionally inactive chromatin, such as lysine nine di- and tri-methylated histone H3 (H3K9me2 and H3K9me3) (Guelen et al., 2008; Wen et al., 2009). The peripheral chromatin immediately adjacent to the nuclear envelope is known as “epichromatin” and can be visualized in cells from different species using a special bivalent mouse monoclonal anti-nucleosome antibody (mAb PL2-6), which binds to the acidic patch on nucleosomes (Olins and Olins, 2018). This suggests that the chromatin close to the nuclear lamina not only has a peculiar composition, but additionally harbors a unique nucleosome structure that is not seen elsewhere in the nucleus. In contrast, more central parts of the nucleus contain chromatin enriched in histone marks of transcriptionally active or permissive chromatin, such as lysine 4 tri-methylated histone H3 (H3K4me3) and lysine 27 acetylated histone H3 (H3K27Ac), which mark iLADs (van Steensel and Belmont, 2017). Although this radial arrangement of chromatin has been observed in many cell types of different species, exceptions also exist. For example, the rod cells of nocturnal animals have the opposite pattern, with more open and active chromatin close to the nuclear lamina, and compact and transcriptionally inactive chromatin amassed in the nuclear center (Solovei et al., 2009). This inverted chromatin arrangement is believed to redirect photons toward the light-sensing outer segments of the rods, thus facilitating vision in darkness (Solovei et al., 2009).

**Figure 1:**
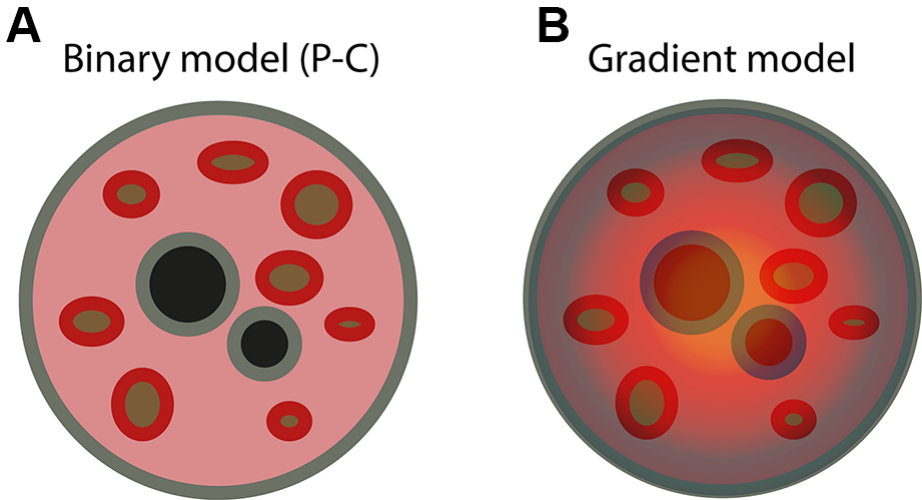
Two different models of radial organization in the mammalian nucleus. **(A)** Binary model of radial nuclear organization (“periphery vs. center” or “P-C” model). According to the P-C model, inactive chromatin (gray) is localized along the nuclear lamina (the “nuclear periphery”) and around nucleoli (black), whereas active chromatin (pink) is distributed in the intervening space (the “nuclear center”), without any specific radial order. Hubs of active chromatin (red) are positioned around speckles (brown), as revealed by TSA-seq (Chen et al., 2018). **(B)** We propose instead a gradient model of radial nuclear organization, according to which both active and inactive chromatin form a continuous gradient along the nuclear radius, with inactive chromatin concentrated near the nuclear lamina and around nucleoli, and active chromatin increasing in concentration toward the nuclear center and around speckles. In the gradient model, every genomic locus has a preferred radial location, which is determined by a “radial ZIP code,” although the exact position can vary from cell to cell. The gradient model also postulates that the content of sub-nuclear structures, such as speckles and nucleoli, as well as the inter-chromatin space, are also radially arranged along a similar gradient.

Altogether, the above observations can be summarized in a simplified binary model of radial chromatin organization, which we name “periphery *vs.* center” or “P-C” model (**Figure 1A**). According to the P-C model, the nuclear periphery consists of largely inactive chromatin domains, such as LADs, B compartments, and their constituent B-TADs, which are localized mainly close to the nuclear lamina, but also around nucleoli. On the other hand, the nuclear center is composed of more active chromatin regions, including iLADs, A compartments, and A-TADs, which are distributed in the remaining nuclear space. Although the P-C model provides a simplified framework for spatially organizing various chromatin domains with distinct functional properties, the division between nuclear periphery and center is rather artificial, because it is not clear where the boundary between the two compartments lies (if there exists one). Furthermore, while it is relatively easy to define the nuclear periphery using the lamina as reference, the definition of the nuclear center is more problematic: strictly geometrically speaking, only spheroidal nuclei have a defined center, while for ellipsoidal nuclei, such as those of fibroblasts, the definition is less clear. This ambiguity in distinguishing between nuclear periphery and center is also highlighted by the fact that the same LADs can be found both close to the nuclear lamina, as well as in more interior parts of the nucleus, around nucleoli (Kind et al., 2013). Indeed, a comparison between LADs identified in human fibroblasts (Guelen et al., 2008) and NADs identified in HeLa cells by sequencing the nucleoli-associated portions of the genome (Németh et al., 2010), showed that LADs tend to overlap with NADs. Thus, although it is clear that different types of chromatin are differentially positioned with respect to the nuclear lamina and peri-nucleolar space, a detailed map of the radial organization of chromatin in the cell nucleus is still missing.

One important limitation of the P-C model described above is that it does not explain how active regions are spatially arranged between nucleoli and the lamina. In an attempt to answer this question, two sequencing-based methods were recently developed: TSA-seq (Chen et al., 2018) and SPRITE (Quinodoz et al., 2018). In TSA-seq, the chromatin proximal to a defined sub-nuclear structure, such as nuclear speckles (Spector and Lamond, 2011), is targeted by a specific antibody and subsequently biotinylated using a tyramide reaction. The amount of biotin that gets covalently bound to DNA decreases exponentially as the distance from the sub-nuclear structure targeted by the antibody increases. By sequencing the resulting biotinylated DNA, the parts of the genome that are in close physical proximity to the targeted sub-nuclear structure can be distinguished from the genomic regions that are farther away (Chen et al., 2018). Using TSA-seq, it was shown that, in K562 human chronic myeloid leukemia cells, transcriptionally active genomic regions tend to form two distinct hubs, one localized in a range of a few nanometers around speckles, and the other more dispersed in the space between speckles and the nuclear lamina (Chen et al., 2018). Unlike TSA-seq, SPRITE measures chromosome contacts as well as DNA-RNA interactions without the use of proximity ligation, in contrast to Hi-C (Quinodoz et al., 2018). Using SPRITE, a hub of inter-chromosomal interactions involving transcriptionally active genes was found to be organized around speckles, whereas inter-chromosomal interactions involving inactive regions were organized around nucleoli, in both mouse embryonic stem and human lymphoblastoid cells (Quinodoz et al., 2018). Although these studies were the first to reveal the importance of sub-nuclear structures in shaping the higher-order spatial organization of chromatin in the nucleus, they still do not answer the question of whether the repertoire of genomic loci that speckles and nucleoli contact varies depending on the radial distance of these sub-nuclear structures from the lamina.

### What Shapes Chromatin Radiality?

The studies summarized above clearly indicate that the nucleus of mammalian cells is characterized by some level of radial organization. However, the forces and molecular mechanisms that shape this radial organization remain largely elusive. The primary reason for this is the fact that, until now, it has been extremely challenging to selectively perturb the radial position of defined genomic regions or even entire chromosomes in a controlled fashion, followed by assessing the effect of such perturbations on the global nuclear architecture. Even more limiting has been the lack of genome-wide methods capable of measuring the distance from the nuclear lamina of thousands of genomic loci simultaneously. So far, the strongest evidence pointing to the existence of specific mechanisms that shape chromatin radiality comes from experiments in which specific protein components of the nuclear lamina were genetically ablated. In post-mitotic mouse cells, simultaneous knockout of lamin A and C isoforms and of the lamin B receptor (LBR)—the three major constituents of the nuclear lamina—led to condensation of heterochromatin in the nuclear interior (Solovei et al., 2013). The resulting pattern was reminiscent of the inverted chromatin arrangement seen in mouse rod cells, which indeed lack expression of both lamin A/C and LBR (Solovei et al., 2013). In mouse embryonic stem cells, loss of lamins caused the detachment of certain LADs from the nuclear lamina, and disrupted 3D chromatin contacts in the nuclear interior (Zheng et al., 2018). Similarly, knockdown of emerin in human primary epidermal keratinocytes—another protein component of the nuclear lamina—resulted in chromosome repositioning inside the nucleus and reduction of H3K9me3 levels and distribution (Le et al., 2016). These findings are in line with the observation that, in humans carrying congenital mutations of lamin genes, the radial location of certain chromosomes is altered, which in turn is associated with changes in gene expression (Malhas et al., 2007; Mewborn et al., 2010; Puckelwartz et al., 2011). In addition to components of the nuclear lamina, histone modifications might also play a role in shaping chromatin radiality. For example, treatment of human adenocarcinoma HT29 cells with a histone deacetylase inhibitor resulted in increased levels of acetylated histone H3K9 and, concurrently, in relocation of multiple loci from the nuclear periphery toward the center (Strasák et al., 2009). Using a different approach combining RNA interference with high-throughput DNA FISH, 50 different factors were found to be involved in determining the radial position of three different genes in hTERT immortalized CRL-1474 human skin fibroblasts (Shachar et al., 2015). These radial positioning factors included chromatin remodelers, histone modifiers, as well as components of the nuclear pore and envelope (Shachar et al., 2015). Although this study assessed the radial position of only three genes, it was the first to establish that multiple factors, in addition to nuclear lamina components, can contribute to the radial arrangement of specific gene loci in the nucleus. Changes in the radial position of a gene locus can also be induced by selectively perturbing its transcriptional activity. For instance, tethering a viral transcriptional activator to a transgene constitutively localized close to the nuclear lamina caused the relocation of the transgene toward the nuclear interior in Chinese hamster ovary cells (Chuang et al., 2006). Similarly, targeting of a transcriptional activation domain to the promoters of genes in facultative LADs modified their radial position, moving them toward the nuclear center in mouse embryonic stem cells (Therizols et al., 2014). Notably, in the same cell type, a local change in chromatin condensation, without transcriptional activation, was sufficient to reposition these genes from the nuclear periphery to the center (Therizols et al., 2014). Thus, it is possible that the landscape of chromatin compaction, coupled with the action of specific tethers such as lamins, act as the primary forces that shape the radial organization of chromatin in the nucleus. However, it cannot be ruled out that transcription *per se* contributes to shape the global arrangement of chromatin, perhaps by locally modulating its compaction. In fact, based on a biophysical model of chromatin folding, it was recently proposed that transcriptional activity, rather than gene density, represents the dominant force that orchestrates the radial arrangement of chromatin in the nucleus (Cook and Marenduzzo, 2018).

In addition to specific nuclear proteins, histone modifications, and transcription, other factors have been suggested to contribute to shaping chromatin radiality. Genome-intrinsic features, such as chromosome size, guanine-cytosine (GC)-content, gene density, as well as the type and density of repetitive elements along the genome have long been associated with the radial arrangement of chromatin in the nucleus, in different human and mouse cell types (Bridger et al., 2000; Boyle et al., 2001; Mayer et al., 2005). Indeed, computer simulations of the formation of CTs at the onset of the G1-phase have suggested that the radial arrangement of chromosomes in interphase nuclei is predominantly dictated by the density of genes along each chromosome (Kreth et al., 2004). Gene density and GC-content have also been related to the topology of individual CTs, as gene-rich chromosomes, such as chr11, 17, and 19, were shown to have a more irregular shape, at least in WI38 human fibroblasts (Sehgal et al., 2014). A potentially important, yet largely neglected, factor that might contribute to dictate how chromatin is radially organized is nuclear shape. The shape and size of the nucleus are ultimately determined by a complex interplay between cytoskeletal forces and chromatin compaction inside the nucleus (Mukherjee et al., 2016). A recent study showed that experimental perturbations of the nuclear geometry in NIH 3T3 fibroblasts resulted in chromosome repositioning and changes in gene expression (Wang et al., 2017). Notably, alterations in nuclear shape and size are a defining feature of cancer cells (Uhler and Shivashankar, 2018), but how exactly chromatin is radially arranged in cancer nuclei with different shapes remains to be investigated.

An emerging concept in the field of genome organization is that hydrophobic interactions between intrinsically disordered domains of certain nuclear proteins, such as transcription factors, can induce liquid-liquid phase separation between different genomic regions, thus contributing to spatially organize chromatin in the nucleus (Cramer, 2019). Similarly, homotypic interactions between certain repetitive elements, such as short interspersed nuclear elements (SINEs) and long interspersed nuclear elements (LINEs), have been proposed to drive the physical separation between euchromatin and heterochromatin in the nucleus (Solovei et al., 2016). Along the same line, a model named “dog-on-a-lead” was proposed, according to which the radial arrangement of chromatin in the nucleus is dictated by repetitive genomic sequences, including ribosomal RNA genes and centromeric repeats (Krijger and de Laat, 2013). Homotypic interactions between DNA repeats might be either direct or mediated by proteins (*e.g.*, chromatin-bound factors and/or histone modifications) or by long non-coding RNAs (lncRNAs). Indeed, several lncRNAs have been implicated in reorganizing genome architecture and initiating the formation of nuclear compartments (Engreitz et al., 2016), including the lncRNA *Xist* that mediates X chromosome inactivation (Jégu et al., 2017).

In addition to phase separation, sub-nuclear compartments, such as nucleoli (Németh and Längst, 2011) and speckles (Spector and Lamond, 2011), have recently come into the spotlight as possible organizers of the higher-order structure of chromatin in the nucleus. As discussed above, hubs of inter-chromosome contacts are formed around nucleoli and speckles (Quinodoz et al., 2018), and most of the actively transcribed genes were found to be localized either around speckles or in the intervening space between them and the nuclear lamina, in both mouse embryonic stem and human lymphoblastoid cells (Chen et al., 2018). In addition to sub-nuclear structures, specific gene loci or clusters of genes might, under certain conditions, contribute to the radial arrangement of chromatin in the nucleus, by acting as “nucleators” that pull transcriptionally active chromatin toward the nuclear interior. For instance, in mouse embryonic stem cells, the pluripotency factors Oct4 and Nanog were shown to organize clusters of pluripotency factor-binding sites, and thus contribute to the unique higher-order chromatin organization of these cells (de Wit et al., 2013). In conclusion, multiple forces are likely in place to shape the final blueprint of radial chromatin organization in a given cell. Teasing apart the relative contribution of different forces and their mechanism of action will, however, require the design of sophisticated experiments, in which each of them is perturbed separately, and the effect on radial organization is quantified genome-wide.

### Toward a Unified Model of Spatial Nuclear Organization

In this review, we have summarized the existing evidence that chromosomes and the underlying sub-chromosomal domains are non-randomly arranged with respect to the nuclear lamina. However, many questions still await an answer before we can reach a thorough understanding of this fundamental aspect of cell biology: *Is chromatin randomly placed between the lamina and nucleoli, or is there a preferred radial location for every gene? If so, can we identify a “radial ZIP code” that specifies where a given locus will be preferentially radially located? Does radial organization only apply to chromatin or also to proteins and RNA in the inter-chromatin space? What are the forces that build and maintain the radial architecture of the nucleus after each cell division? Is radial nuclear organization disrupted by genomic rearrangements that occur, for instance, during tumorigenesis? If so, does disruption of radial nuclear organization contribute to cancer progression?* Answering these questions will require drawing comprehensive maps of radial nuclear organization in many cell and tissue types, as well as in different disease conditions.

The P-C model described above (**Figure 1A**) recapitulates many of the observations on radial chromatin organization that have been reported so far. However, one key limitation of this model is that it does not specify whether any locus along a given genome has a defined probability of being located at a specific radial location, or whether it is either peripherally (close to the lamina) or centrally located. We propose instead a gradient model of spatial nuclear organization, according to which every genomic locus has a preferred radial location (a “radial ZIP code”) that is dependent on the type, differentiation, and functional state of the cell in which it is present (**Figure 1B**). This model does not only apply to DNA, as we envisage that the entire inter-chromatin space may also be radially organized, with different probabilities of finding specific protein complexes, lncRNAs, and nuclear bodies at defined radial positions. Notably, a gradient model of chromatin organization was already proposed 26 years ago, based on how the radial arrangement of chromatin in the nucleus changes during chondrogenesis in chicken embryos (Erenpreisa and Zhukotsky, 1993). Testing the validity of the gradient model, which we propose here, will require developing new methods that can probe the radial location of DNA, RNA, and proteins throughout the nuclear space, and not just close to the nuclear lamina, as done by lamin DamID (Guelen et al., 2008). The recent development of SPRITE (Quinodoz et al., 2018) and TSA-seq (Chen et al., 2018) are important steps in this direction, and, in our lab, we are also developing new methodologies for probing the radial position of genomic loci and proteins all along the nuclear radius. Ideally, these methodologies should, one day, be able to probe genome-wide radial locations in single cells, allowing us to quantify the extent of cell-to-cell variability in nuclear radial organization, and relate it to gene expression variability. In addition to developing novel methodologies, testing the gradient model that we have proposed here, will require devising ways to experimentally perturb the radial location of large chromatin domains, and possibly entire chromosomes, by changing their sequence, epigenetic status, or transcriptional activity, in a controlled fashion. Finally, we will need to develop spatially resolved methods enabling us to explore chromatin organization directly in cells embedded in their natural tissue context, in order to fully understand how different tissue and organ architectures cross-talk with organization within the cell nucleus. Whatever it takes to get there, we anticipate that obtaining a comprehensive portrait of the radial architecture of the cell nucleus will bring us closer to fully understand how this essential cellular organelle functions in health and disease.

## Author Contributions

Both authors equally contributed to writing this mini-review.

## Funding

This work was supported by grants from the Swedish Research Council (521-2014-2866), the Swedish Cancer Research Foundation (CAN 2015/585), the Ragnar Söderberg Foundation (Fellows in Medicine 2016), and the Strategic Research Programme in Cancer (StratCan) at Karolinska Institutet to NC; and by grants from the Science for Life Laboratory, the Karolinska Institutet KID Funding Program, the Swedish Research Council (621-2014-5503), the Human Frontier Science Program (CDA-00033/2016-C), the Ragnar Söderberg Foundation (Fellows in Medicine 2016), and the European Research Council under the European Union’s Horizon 2020 research and innovation programme (StG-2016_GENOMIS_715727) to MB.

## Conflict of Interest

The authors declare that the research was conducted in the absence of any commercial or financial relationships that could be construed as a potential conflict of interest.
